# TLR4 signaling drives mesenchymal stromal cells commitment to promote tumor microenvironment transformation in multiple myeloma

**DOI:** 10.1038/s41419-019-1959-5

**Published:** 2019-09-20

**Authors:** Cesarina Giallongo, Daniele Tibullo, Giuseppina Camiolo, Nunziatina L. Parrinello, Alessandra Romano, Fabrizio Puglisi, Alessandro Barbato, Concetta Conticello, Gabriella Lupo, Carmelina Daniela Anfuso, Giacomo Lazzarino, Giovanni Li Volti, Giuseppe Alberto Palumbo, Francesco Di Raimondo

**Affiliations:** 1 Section of Haematology, Department of General Surgery and Medical-Surgical Specialties, University of Catania, Catania, Italy; 2 Department of Biomedical and Biotechnological Sciences, University of Catania, Catania, Italy; 3 EuroMediterranean Institute of Science and Technology, Palermo, Italy; 4 Institute of Biochemistry and Clinical Biochemistry, Catholic University of Rome, Largo F. Vito 1, 00168 Rome, Italy; 5 Department of Medical, Surgical Sciences and Advanced Technologies “G. F. Ingrassia”, University of Catania, Catania, Italy

**Keywords:** Myeloma, Translational research

## Abstract

Inflammation represents a key feature and hallmark of tumor microenvironment playing a major role in the interaction with mesenchymal stromal cells (MSC) in cancer progression. The aim of the present study was to investigate the crosstalk between MSCs and myeloma cells (MM) in the pro-inflammatory microenvironment promoting immune evasion and tumor growth. MSC were collected from patients with diagnosis of MGUS (*n* = 10), smoldering myeloma (*n* = 7), multiple myeloma at diagnosis (*n* = 16), relapse (*n* = 5) or refractory (*n* = 3), and from age-matched healthy controls (HC, *n* = 10) and cultured with peripheral blood mononucleated cells (PBMC) from healthy volunteer donors. Similarly to MM, we showed that MSC from smoldering multiple myeloma (SMM) patients activated neutrophils and conferred an immunosuppressive and pro-angiogenic phenotype. Furthermore, co-cultures of plasma cells (PC) and HC-MSC suggested that such activation is driven by MM cells through the switching into a pro-inflammatory phenotype mediated by toll-like receptor 4 (TLR4). These results were further confirmed using a zebrafish as an immunocompetent in vivo model, showing the role of MM–MSC in supporting PCs engraftment and Th2 response. Such effect was abolished following inhibition of TLR4 signaling in MM–MSC before co-injection with PC. Moreover, the addition of a TLR4 inhibitor in the co-culture of HC-MSC with MM cells prevented the activation of the pro-tumor activity in PC-educated MSC. In conclusion, our study provides evidence that TLR4 signaling plays a key role in MSC transformation by inducing a pro-tumor phenotype associated with a permissive microenvironment allowing immune escape and tumor growth.

## Introduction

Multiple myeloma (MM) is characterized by clonal expansion of malignant PC in the bone marrow (BM), monoclonal protein in the blood or urine and clinical symptoms including anemia, bone destruction, and kidney injury^1^. It is a multistep progressing disease starting with an asymptomatic monoclonal gammopathy of undetermined significance (MGUS) through smoldering myeloma (SMM), up to the most aggressive, symptomatic MM^2^. Although overall survival has been significantly improved by new pharmacological agents, such as proteasome inhibitors, immunomodulatory drugs, and monoclonal antibodies, primary and acquired resistance remain a major issue limiting the clinical efficacy of these drugs.

Several lines of evidence suggest that myeloma cells survival, proliferation and chemoresistance strictly depend on the BM niche^3^ and therefore this site may represent an important target for new pharmacological agents. In particular, MSC represent a key component of BM microenvironment playing a close interplay with myeloma cells and, even in the absence of molecular modifications, they can acquire functional abnormalities after the inflammatory process developed during the course of the disease and thus playing a major role in the tumor niche formation^4^. To this regard, previous reports showed that MSC are able to promote tumor development, metastasis and chemoresistance by enhancing tumor growth, angiogenesis and immune escape via an interaction with the immune cells creating an immunosuppressive environment^5^. As far as concern BM, primary alterations of the mesenchymal niche can induce myelodysplasia and acute myeloid leukemia driving the so called niche-driven oncogenesis^6^.

Targeting the stroma is emerging as a new strategy in various types of cancer, such as pancreatic cancer^7^, breast cancer^8^, osteosarcoma^9^, melanoma, and ovarian cancer^10^. In particular, in breast cancer, MSC protect tumor cells by increasing regulatory T cells (Treg) and decreasing the activity of natural killer cells and cytotoxic T lymphocytes, which are responsible for tumor cells killing^11^. Previous studies suggested that inflammation represents a key feature and hallmark of tumor microenvironment and should be taken into due account in the complex pathophysiologic process of cancer progression. Physiologically, inflammation is considered a protective reaction in response to various stimuli and promoting tissue wound healing^4,11^. However, chronic inflammation within the tumor niche promotes cancer growth and immune destruction by mobilization of myeloid-derived suppressor cells (MDSC) and tumor-associated macrophages^12^. Tumor-promoting inflammation is driven by pattern recognition receptors (PRR), such as Toll-like receptors (TLR), involved in the recognition of various damage-associated molecular patterns (DAMP)^13^. TLR are type-I transmembrane glycoproteins that recognize “danger” signals leading to profound cellular and systemic responses that mobilize innate and adaptive host immune cells^14^. Interestingly, previous studies suggested a possible correlation between the stimulation of specific TLR and the activation status of MSC. These cells express several TLRs, which are involved in cell migration, invasion and secretion of immune modulating factors^15^. Specifically, human MSC can be polarized by downstream TLR signaling into two distinct phenotypes defined MSC1 and MSC2^16^. In particular, TLR4-primed MSC, or MSC1, mostly elaborate pro-inflammatory mediators, whereas TLR3-primed MSC, or MSC2, mainly secrete immunosuppressive cytokines. Recently, Lu et al.^17^ demonstrated that TLR4 + MSC from patients with acute myeloid leukemia and lung cancer are able to inhibit NK cell function, thus triggering immune escape pathways. Concerning this, we have previously demonstrated that MM–MSC have a tumor-promoting potential, mediated, at least in part, by their ability to drive the activation of immature myeloid cells in granulocyte-like MDSCs with immunosuppressive activity^18^. Therefore, we hypothesize that such MM–MSC tumor-promoting function may be associated to TLR activation.

The aim of this work is to investigate the existing crosstalk between MSC and myeloma cells in order to identify molecular pathways involved in the pro-inflammatory microenvironment that promote immune evasion and tumor growth.

## Materials and methods

### In vitro analyses

#### MSC collection and culturing conditions

After written informed consent (Azienda ospedaliero Universitaria Policlinico-Vittorio Emanuele, #34/2013/VE), BM samples were collected from patients with diagnosis of MGUS (*n* = 10), smoldering myeloma (*n* = 7), multiple myeloma at diagnosis (*n* = 16) or relapse (*n* = 5) or refractory (*n* = 3), and age-matched healthy controls (HC, *n* = 10). Clinical data of patients included in this study are shown in Table 1.

**Table 1:** Baseline clinical characteristics of patients included in the study and refractory MM patients

	MGUS (*n* = 10)	Newly-diagnosed MM (*n* = 11)	Relapsed MM (*n* = 5)
*Median age (range)*	67 (49–70)	65 (45-68)	67 (38–75)
Males/Females	7/3	7/4	3/2
*Isotype, n*			
IgG	0	8	2
IgA	10	2	2
Light-chain only	0	1	1
*Cytogenetics, n*			
Normal	8	5	1
*del 13*	1	1	2
*del 17*	0	3	0
*t(4;14)*	0	1	2
not performed/failed	1	1	0
*Haemoglobin, g/dl (range)*	12.8 (12–14.5)	10.6 (6.5–13.8)	9.8 (6.6–12.8)
Paletelets 1000/μL (range)	219 (180–315)	221 (90–384)	123 (43–225)
*Bone marrow plasmocytosis >50%, n (%)*	0	5	3
C-reactive protein median, mg/l (range)	0.1 (0.01–4)	4.4 (0.001–8.5)	5.3 (0.05–9.6)
*LDH median, mm/h*	195 (132–213)	209 (109–708)	240 (125–368)
ESR median, mm/h	17 (0–26)	72 (6–134)	84 (10–138)
*STAGE ISS, n*			
1	N.A.	3	0
2	N.A.	5	4
3	N.A.	3	1

Summary of refractory MM patients			
Sex	Age	Type	Number of treatment lines
F	72	IgA-λ	3
M	72	IgG-κ	2
F	64	IgG-κ	1

BM mononuclear cells were obtained after density gradient centrifugation on Ficoll and cultured in Dulbecco’s modified Eagle’s medium (DMEM, Life Technologies, Milan, Italy) supplemented with 10% heat-inactivated FBS, 100 U/ml penicillin, 100 mg/ml streptomycin and 1% l-glutamine. After 3 days culture, non-adherent cells were removed, whereas MSC were selected by their adherence to the plastic plates. Cultures were maintained at 37 °C and 5% CO_2_ and expanded until the third or fourth passage. Selected MSC from patients and HC at the third passage were also tested for MSC specific surface antigen expression using combinations of the following monoclonal antibodies: anti-CD34-ECD (clone 581), anti-CD90-FITC (clone F15.42.1.5), anti-CD105-PE (clone 1G2) and anti-CD45-PC5 (clone J.33) (Beckman coulter, Brea, California, United States). The appropriate isotopic control was also included. Labeled MSC were acquired using a Beckman Coulter FC-500 flow cytometer.

#### Cell cultures

MM cell lines were cultured in RPMI-1640 with 10% (MM1S, H929, OPM2) or 20% (U266) FBS and 1% penicillin-streptomycin. Commercially available stromal cell lines HS-5 were grown in DMEM supplemented with 10% FBS. Human Microvascular Endothelial Cells (HMEC) (Innoprot, Elexalde Derio, Spain) were grown in monolayer in EBM supplemented with 5% fetal bovine serum (FBS), 1% endothelial cell growth supplement (ECGS), 100 U/mL penicillin and 100 mg/mL streptomycin. For the experiments, serum was reduced at 1% v/v.

#### Induction and evaluation of neutrophils polarization

Immature neutrophils are present in the peripheral blood mononucleated cells (PBMC) fraction^19^. PBMC were isolated from healthy volunteer donors after density gradient centrifugation on Ficoll and were cultured alone or co-cultured with MSC derived from healthy subjects or patients with MGUS, SMM or MM (1:100 ratio)^20,21^. MSC were seeded to achieve confluence by 7 days. After one week, PBMC were collected and neutrophils (ed-N), were isolated using anti-CD66b magnetic microbeads (Miltenyi Biotec, Bergisch Gladbach, Germany). Cultures purity and viability were confirmed by cytofluorimetric analysis (Supplementary Fig. 1).

The immunosuppressive capacity of ed-N was analyzed by co-culturing them with autologous CFSE-labeled T cells (1:2) stimulated with 5 μg/mL phytohemagglutinin (PHA). T cells were isolated by magnetic cell separation using human CD3 microbeads (Miltenyi Biotec). T lymphocytes labeling was performed following incubation at 37 °C for 20 min in 1 ml PBS containing 1 μM CFSE. Controls included a positive T cell proliferation control (T cells treated with PHA) and a negative one (untreated T cells). After three days, T cell proliferation was measured by CFSE dilution and analyzed by flow cytometry.

Bortezomib (BTZ, 5 nM), lenalidomide (LENA, 10 μM) and pomalidomide (POMA, 1 μM)^22–24^ were added during co-culture of PBMC with MM-MSC (from patients at diagnosis or relapsed or refractory to therapies) to investigate the effects of the most commonly used anti-myeloma drugs in priming MSC-mediated neutrophils activation.

Tube formation assay: ed-N were co-cultured with HMEC (1:2) to evaluate their pro-angiogenic capacity. Cells were seeded in 96-well plates covered with polymerized growth factor-reduced Matrigel matrix (BD, Franklin Lakes, NJ, USA), incubated for 4 h (37 °C, 5% CO_2_) and photographed at ×100 magnification using an inverted Leica DM IRB microscope equipped with a Charge-Coupled Device (CCD) camera, as previously described^25^.

#### Induction of a polarized phenotype in HC-MSC

HC-MSC and HS-5 were pre-treated with 10 ng/mL Lipopolysaccharide (LPS) (Sigma-Aldrich, Milan, Italy) or 1 μg/mL Poly I:C (Polyinosinic:polycytidylic acid, Sigma-Aldrich) for 24 h. After washing, stromal cells were then cultured with PBMC. In a separate set of experiments, HC-MSC or HS-5 cells were incubated with human MM cell lines for 24 h (1:10) before co-culturing with PBMC. In order to assess TLR4 involvement under our experimental conditions, cultures were also treated with TAK-242 (Sigma-Aldrich), a blocker of signaling transduction triggered by TLR4 intracellular domain. For PC and HS-5 co-cultures, we used 10 μM TAK-242 2 h before adding MM cells. TAK-242 treatment did not affect HS-5 viability (Supplementary Fig. 2).

#### RT-qPCR

After RNA extraction and reverse transcription^26^, ed-N were analyzed for expression of ARG1, NOS2 and TNFα mRNA. Their expression was assessed by using 7900HT Fast Real-Time PCR System and TaqMan Universal PCR Master Mix (ThermoFisher, Monza, Italy). For each sample, the relative expression level of each studied mRNA was normalized using GAPDH as invariant control.

#### Western blot analysis

Briefly, for western blot analysis 50 μg of proteins were loaded onto a 12% polyacrylamide gel Mini- PROTEAN® TGXTM (BIO-RAD, Milan, Italy) followed by electrotransfer to nitrocellulose membrane Trans- Blot® TurboTM (BIO-RAD) using Trans- Blot® SE Semi-Dry Transfer Cell (BIO-RAD). Subsequently, membrane was blocked in Odyssey Blocking Buffer (Licor, Milan, Italy) for 1 h at room temperature. After blocking, membrane was washed three times in PBS for 5 min and incubated with primary antibodies against human MyD88, TLR4 and β-actin (Santa Cruz Biotechnology, Santa Cruz, CA, USA), overnight at 4 °C. Next day, membranes were washed three times in PBS for 5 min and incubated with Infrared anti-mouse IRDye800CW (1:5000) and anti-rabbit IRDye700CW secondary antibodies (1:5000) in PBS/0.5% Tween-20 for 1 h at room temperature. All antibodies were diluted in Odyssey Blocking Buffer. The blots were visualized using Odyssey Infrared Imaging Scanner (Licor, Milan, Italy) and protein levels were quantified by densitometric analysis of antibody responses. Data were normalized to protein levels of β-actin.

#### Immunofluorescence

Cells were grown directly on chamber slides before immunofluorescence. After washing with phosphate-buffered saline (PBS), cells were fixed in in 4% paraformaldehyde for 20 min at room temperature. After fixation, cells were washed three times in PBS for 5 min. Subsequently, cells were incubated with primary antibody against IRF3 (anti-rabbit; Santa Cruz Biotechnology) and NFKB (anti-mouse; Santa Cruz Biotechnology) at dilution 1:100, overnight at 4 °C. Next day, cells were washed three times in PBS for 5 min and incubated with secondary antibodies: TRITC (anti-rabbit) and FITC (anti-mouse) at dilution 1:200 for 1 h at room temperature. The slides were mounted with medium containing DAPI (4,6-diamidino-2-phenylindole) to visualize nuclei. The fluorescent images were obtained using a Zeiss Axio Imager Z1 Microscope with Apotome 2 system (Zeiss, Milan, Italy).

### In vivo zebrafish model of human MM xenograft

#### Ethics statement

The experimental procedures were approved by the Animal Studies Committee of Ministero della Salute Italy (Approval code: 813/2017-PR, 23 October 2017). All of the procedures were performed according to the relevant regulations.

#### Zebrafish husbandry

Adult wildtype (5–8-months-old) AB zebrafish were used for this study. Fishes were housed at a density of five fishes per tank in mixed-sex groups in 2.5 L tanks on a recirculating system in 28 °C water in a room with a 14:10 h light:dark cycle. System water was carbon-filtered municipal tap water, filtered through a 20 μm pleated particulate filter, and exposed to 40 W UV light [35]. Standard feeding protocol was three meals daily of Tetra-Min (Tetra) in the CAPIR (University of Catania) facility.

#### Xenotransplantation procedure

Zebrafish were anesthetized with 0.02% tricaine (Sigma-Aldrich) and injected with U266 cells mixed with HC- or MM-MSC in a 1:1 ratio^27,28^ in PBS (5 × 10^4^/5 × 10^4^) using a borosilicate glass capillary and a microinjector system.

Prior to implantation, MM cells were labeled with DiIC18(5)-DS (1,1′-Dioctadecyl-3,3,3′,3′-Tetramethylindodicarbocyanine-5,5′-Disulfonic Acid) (ThermoFisher) at a final concentration of 1 μM for 5 min at 37 °C in a 5% CO2 atmosphere and 15 min at 4 °C. We evaluated the tumor xenografts, by Multispectral VIS-NIR Fluorescence Imaging (MS-FLI) in vivo Xtreme II-Bruker (Bruker, Milan, Italy), at 6 days post-injection and tumor area was measured by ImageJ software^29^.

TLR4 inhibition: MM–MSC were pre-treated in vitro with 5 μM TAK-242 2 h before implantation. After washing, TAK-242 pre-treated MM-MSC were mixed with U266 cells and injected in vivo. We evaluated the tumor xenografts at 3 days post-injection. TAK-242 treatment did not affect MM-MSC viability (Supplementary Fig. 2).

#### Detection of hCD138+ infiltration in tumor xenografts

MM cell xenograft analysis was evaluated by flow cytometry. Wild-type adult zebrafish were anaesthetized, and the kidney/marrow and abdominal organs were dissected and placed in PBS as described^30^. Single cell suspensions were generated by passing through a nylon mesh and stained with propidium iodide (Sigma) to exclude dead cells. Staining with the human monoclonal antibodies against CD138 was performed. This human mAb did not show cross-reactivity with Zebrafish cells (Supplementary Fig. 3). Single cell suspensions from zebrafish were incubated with fluorescently labeled anti-CD138 PE (clone B-A38) antibody (Beckman coulter, Brea, California, United States). After incubation for 15 min at room temperature and subsequent centrifugation, labeled samples were acquired using a Beckman Coulter FC-500 flow cytometer (Beckman Coulter FC-500 flow cytometer). For each sample analyzed, the appropriate isotopic control was also included.

#### RT-qPCR

Total RNA was extracted from kidney/marrow and abdominal organs by Trizol reagent. After reverse transcription, we evaluated expression of the following zebrafish mRNA: zTBX21, zGATA3, zINFγ, zIL4, zIL13. Real-time PCR was performed by using 7900HT Fast Real-Time and Brilliant III Ultra-Fast SYBR Green QPCR Master Mix (Agilent Technologies). Primer sequences are reported in Supplementary Fig. 4. For each sample, the relative expression level of each studied mRNA was normalized using zGAPDH as invariant control.

#### Statistical analysis

The data are expressed as mean ± SD. Statistical analysis was carried out by Student’s *t*-test, ANOVA test. A *p* value < 0.05 was considered to indicate a statistically significant difference between experimental and control groups.

## Results

### SMM- and MM-MSC, but not HC- and MGUS- MSC, drive neutrophils toward an immunosuppressive and pro-angiogenic phenotype

**Fig. 1:**
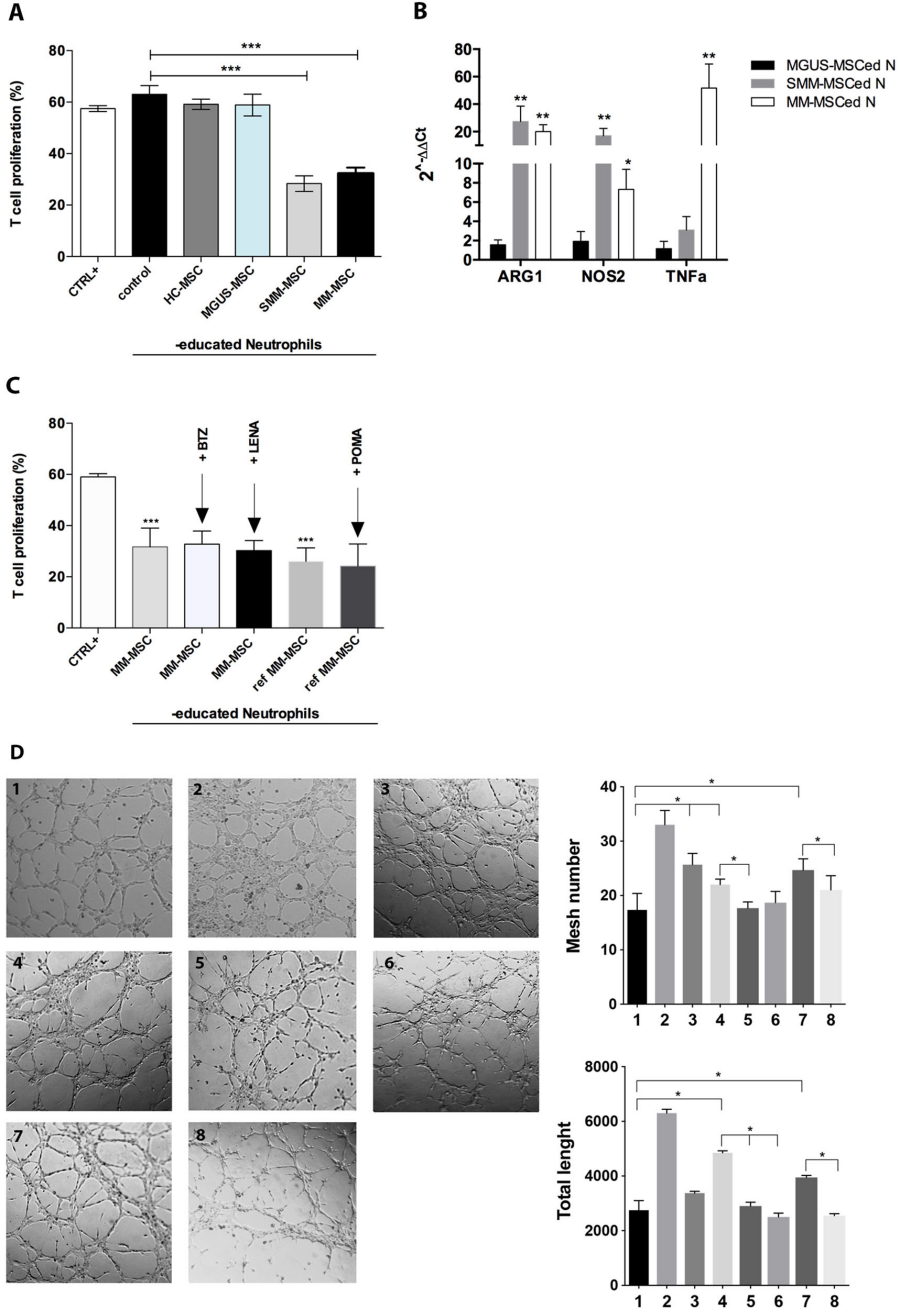
SMM- and MM-MSC activated neutrophils in immunosuppressive and pro-angiogenic cells. **a** Only SMM- and MM-MSCed-N exhibited suppressive effects compared to N control (isolated from PBMC cultured without MSC). CTRL+ : T lymphocytes incubated only with PHA. **b** Before incubation with T cells, ed-N were analyzed for the expression of immune modulatory factors. Calculated value of 2^−ΔΔCt^ in control (HC-MSC educated-neutrophils) was 1. **c** Adding BTZ, LENA or POMA during co-culture of PBMC with MM-MSC, isolated neutrophils did not lose immunosuppressive ability. *Ref MM-MSC:* MSC isolated from patients with refractory MM. **d** HMEC were plated on Matrigel in the absence (1, control) or presence of VEGF-A (2, positive control), of SMM-MSCed-N (3), MM-MSCed-N (4), MM-MSCed-N isolated from co-culture with BTZ (5) or LENA (6), refractory MM-MSCed-N (7) or refractory MM-MSCed-N isolated from co-culture with POMA (8). After 5 h, SMM-MSCed-N and refractory MM- and MM-MSCed-N induced tube formation. The pro-angiogenic effect was significantly reduced by the proteasome inhibitor and the immunomodulatory drugs. *p < 0.05; **p < 0.01; ***p < 0.001

In order to determine whether MGUS- and SMM-MSC have the same immunological alteration as MM-MSC^18^, PBMC from healthy donors were co-cultured with HC-, MGUS-, SMM- or MM-MSC for one week. After magnetic cell separation, we found that only neutrophils cultured with SMM-MSC (SMM-MSCed-N) were able to suppress T cell proliferation (*p* < 0.0001) similarly to MM-MSCed-N (Fig. 1a). We observed no reduction of T cell proliferation following incubation of T lymphocytes with MGUS-MSCed-N or HC-MSCed-N. Consistently, SMM- and MM-MSCed-N significantly up-regulated ARG1, NOS2 and TNFα (Fig. 1b). In order to further evaluate whether commonly used drugs for MM were able to revert MM-MSC-mediated neutrophils activation, we treated co-cultures of PBMC with MM-MSC from patients at diagnosis or with refractory MM with BTZ, LENA or POMA. These sets of experiments showed that neutrophils educated in co-culture with MM–MSC in presence of these drugs did not lose their immunosuppressive ability (Fig. 1c).

Our in vitro results also showed the pro-angiogenic ability of SMM- and MM-MSCed-N with an increase of both tube length and mesh number compared to control (isolated from PBMC cultured in medium alone) (*p* < 0.05) (Fig. 1d). The number of the interconnections between the tubes provides information about the way the HMEC organize them and grow. HREC clusters emitted long offshoots from cell bodies; the emission of such processes (branch points) is essential for HMEC in order to mutually come in physical contact, necessary for their recognition and spatial organization. Interestingly, we also showed that the pro-angiogenic capacity was abolished when neutrophils were exposed to BTZ, LENA or POMA during co-culture with MM-MSC.

### MM cells promote MSC commitment toward an inflammatory status

**Fig. 2:**
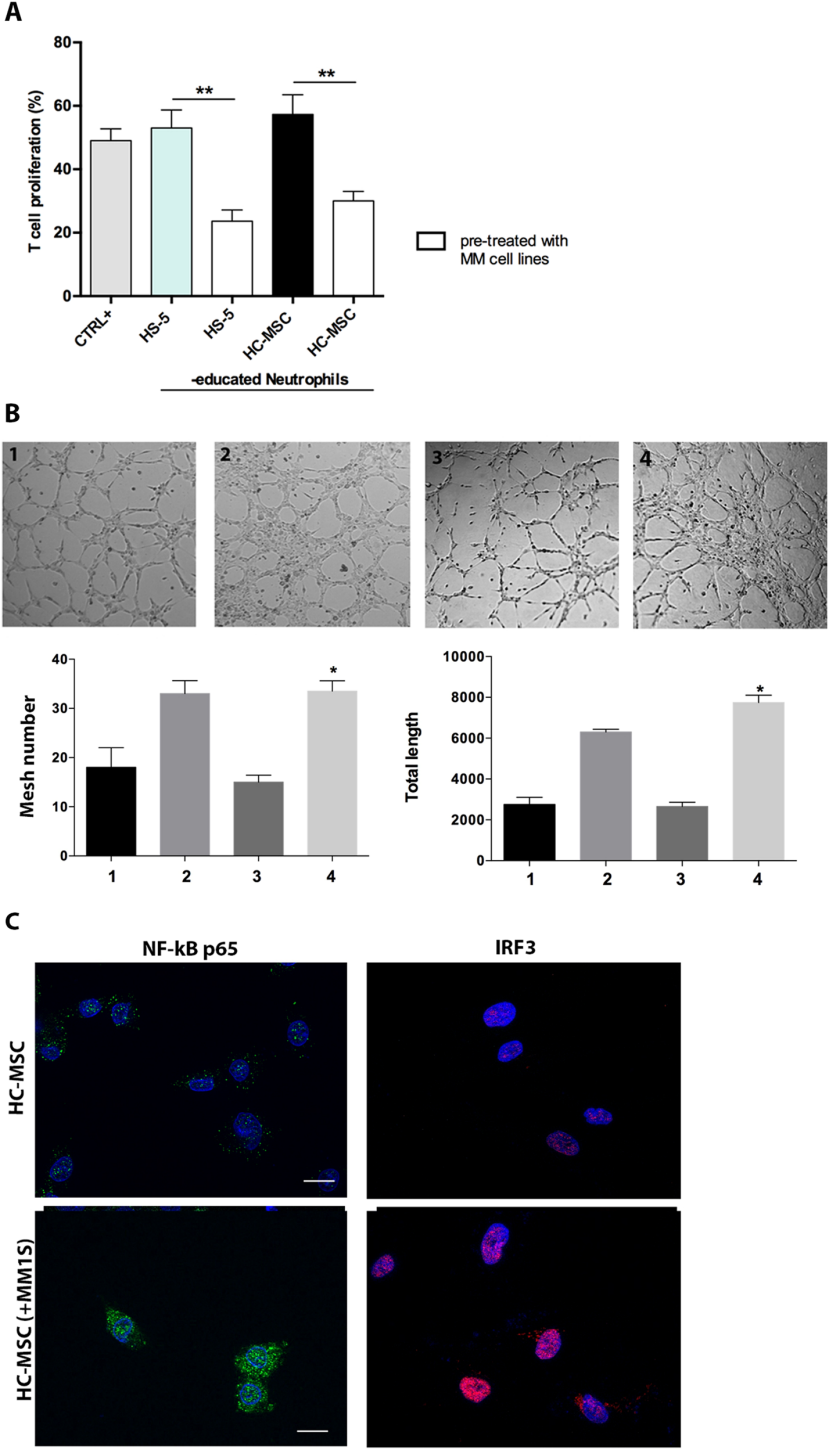
PC activated MSC towards an inflammatory phenotype. **a** Neutrophils isolated from co-culture with HS-5 or HC-MSC 24 h before pre-treatment with MM cells were able to inhibit T cell proliferation (**b**) and showed pro-angiogenic capacity in vitro. 1: HMEC control; 2: HMEC in presence of VEGF-A (positive control); 3: plus HC-MSCed-N; 4: plus HC-MSCed-N isolated from co-culture with HC-MSC pre-treated with MM cells. Magnification ×100. **c** Detection of NF-kB and IRF3 nuclear translocation was performed by incubation respectively with anti-mouse and anti-rabbit monoclonal antibodies followed by secondary antibodies conjugated to FITC (green) or TRITC (red). Counterstaining of cells was performed by using the nuclear dye, DAPI (blue). The photographs result from sequential analysis of the same microscopic field, followed by merging of different images with specific staining. *p < 0.05; **p < 0.01

To address whether PC are responsible for the immune alteration observed in SMM- and MM-MSC, we pre-treated HS-5 cell line or HC-MSC with MM cell lines for 24 h before co-culturing with PBMC. PC pre-treatment drove MSC from healthy controls to activate neutrophils in immunosuppressive and pro-angiogenic cells, similarly to SMM- and MM-MSC (Fig. 2a, b). Indeed, neutrophils educated by PC pre-treated MSC were able to inhibit T cell proliferation (*p* < 0.01) and increased both tube length and mesh number after incubation with endothelial cells (*p* < 0.05). Since MSC can be polarized by downstream TLR signaling into pro-inflammatory MSC1 or immunosuppressive MSC2, we further analyzed the two TLR4 and TLR3 downstream pathways, NF-κB and IRF3 (Interferon regulatory factor 3), in HC-MSC after co-culture with PC. An early NF-κB (starting after 6 h) and a late IRF3 (at 24 h) nuclear translocation was observed (Fig. 2c).

### TLR4 signaling activates healthy MSC and induces the same functional alteration of MM-MSC

**Fig. 3:**
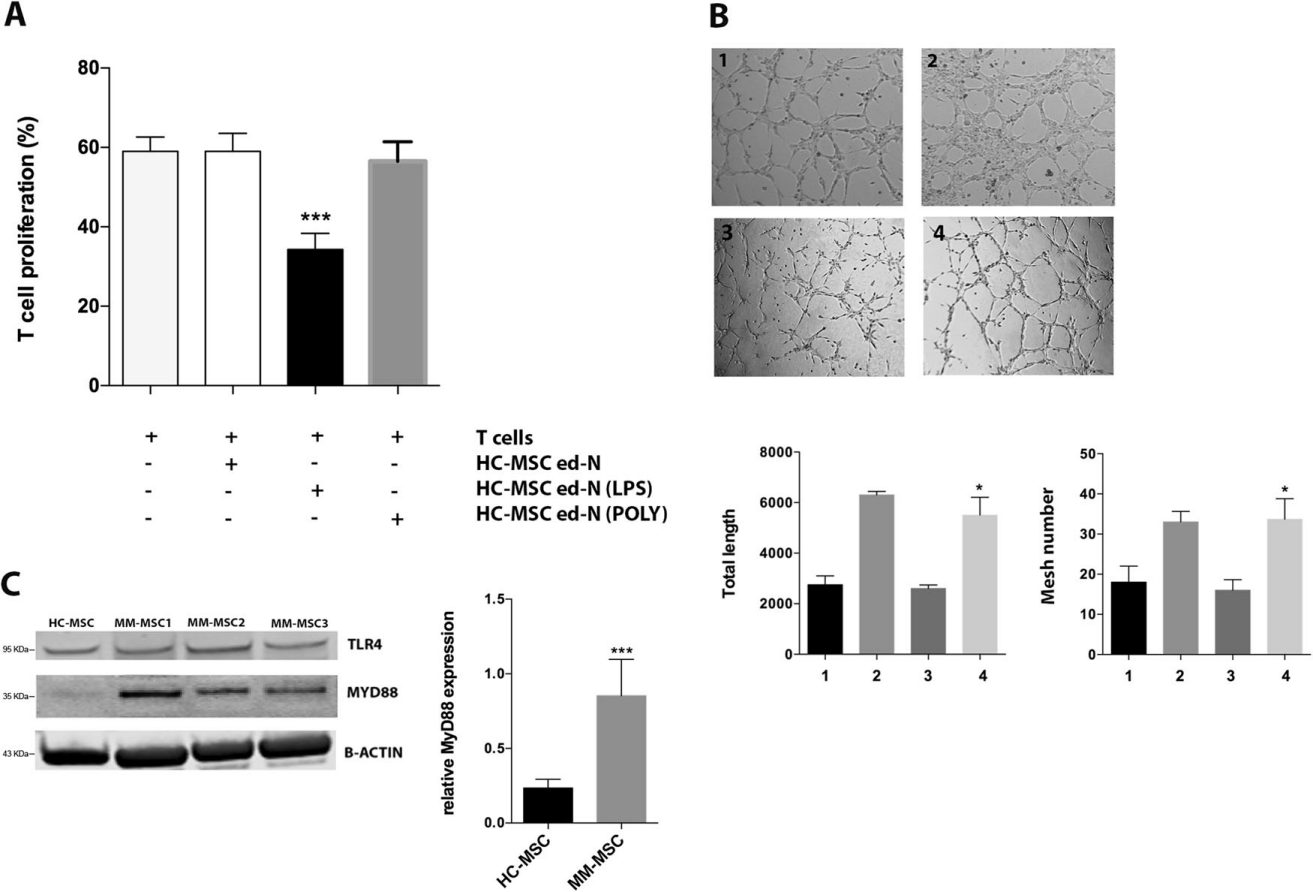
LPS induced the same immunological alteration of MM-MSC in healthy MSC. **a** Neutrophils isolated from co-culture with HC-MSC pre-treated with LPS were able to inhibit T cell proliferation. **b** After the pre-treatment with LPS, HC-MSC educated-neutrophils showed pro-angiogenic capacity in vitro. 1: HMEC control; 2: HMEC in presence of VEGF-A (positive control); 3: HC-MSCed-N; 4: plus ed-N isolated from co-culture of PBMC with HC-MSC pre-treated with LPS. Magnification ×100. **c** MyD88 expression was increased in MM-MSC (*n* = 6) compared to HC-MSC (*n* = 3). For western blotting analysis, the optical density of the bands was measured using Scion Image software. All showed results represent the means of four independent experiments; error bars denote SD. *p < 0.05; ***p < 0.001

Next, HC-MSC were pre-treated with LPS or poly(I:C) as agonists of TLR4 and TLR3 respectively. After 24 h, HC-MSC were then cultured with healthy PBMC. Only LPS pre-treatment activated MSC in stromal cells able to educate neutrophils into immunosuppressive cells (*p* < 0.001) (Fig. 3a). No effects were observed after TLR3 stimulation. We also investigated the ability of LPS pre-treated MSC to activate neutrophils in pro-angiogenic cells. As shown in Fig. 3b, these ed-N were able to increase both the mesh number and the total tube length (*p* < 0.05) after incubation with HMEC. Subsequently, we analyzed whether TLR4 was up-regulated in MM-MSC. Western blotting analysis did not show any change in TLR4 protein levels between HC-MSC and MM-MSC, but we observed the up-regulation of its adaptor MyD88, which is an important contributing protein in the TLR4 (but not in the TLR3) signaling cascade (*p* < 0.001; Fig. 3c).

### SMM- and MM-MSC favor tumor engraftment and immune escape in vivo

**Fig. 4:**
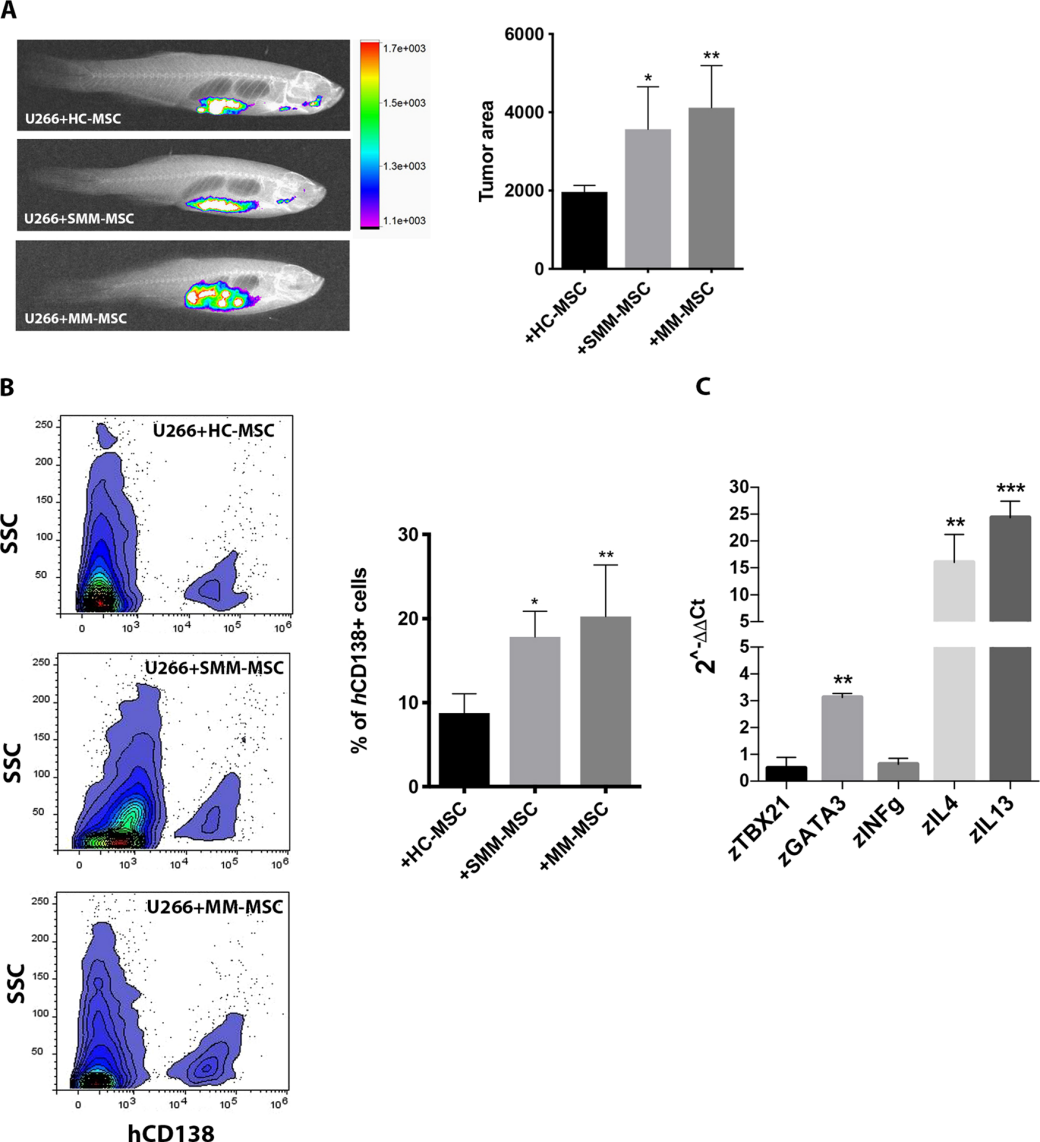
MM-MSC and SMM-MSC enhanced PC engraftment and promoted immune escape mechanisms in zebrafish. Three animals were engrafted for every combination of PC with SMM-MSC (*n* = 3), MM-MSC (*n* = 8) or HC-MSC (*n* = 4). **a** Evaluation of tumor xenografts was performed by tomography after 6 days. **b** Detection of human MM cells using flow cytometry. **c** Analysis of changes in the Th1/Th2 balance in zebrafish using real time PCR. Results for animals co-injected with PC and MM-MSC are shown (compared to animals injected with PC and HC-MSC); calculated value of 2^−ΔΔCt^ in zebrafish injected with U266 and HC-MSC was 1. All analyses were performed 6 days after cellular mixture injection. *p < 0.05; **p < 0.01; ***p < 0.001

We further investigated the pro-tumor behavior of SMM- and MM-MSC in vivo. Six days after xenotransplantation of fluorescent-labeled MM cells mixed with HC-, SMM- or MM-MSC, we observed that zebrafish co-injected with PC and SMM- or MM-MSC showed enhanced MM cell engraftment compared with animals injected with PC and HC-MSC used as controls (respectively *p* < 0.05 and *p* < 0.01; Fig. 4a). Furthermore, cell suspension from the kidney/marrow and abdominal organs was analyzed by flow cytometry to quantify MM cells engraftment. Indeed, bioinformatic approach by using ENSEMBL revealed that zebrafish has not ortholog genes of human CD138 thus allowing the use of *h*CD138 antibody to identify human myeloma cells. Obtained data revealed 9% (*p* = 0.027) and 11% (*p* = 0.005) more *h*CD138+ in zebrafish injected respectively with PC and SMM-MSC or MM-MSC compared to control group (Fig. 4b). Similarly to mice and humans, also zebrafish has a genetically defined Th1/Th2 bias^31^. Therefore, we examined the expression of the master regulator transcription factors for Th1/Th2 and Th1- and Th2-type cytokines to better assess in vivo the involvement of the immune escape mechanisms promoted by co-injection of PC with MM-MSC. To evaluate Th1/Th2 balance we analyzed mRNA levels of *z*TBX21 (T-box transcription factor 21), a Th1 cell transcription factor important for Th1 lineage commitment and *z*GATA3, a well-known regulator of Th2 cell differentiation. In addition, we also evaluated the expression of *z*IL-4 (*z*IL4b) and *z*IL-13 (Th2-type cytokines) and *z*IFN-γ (*z*INFγ1-2) (a Th1-type cytokine) genes^32,33^. We found that *z*GATA3, *z*IL-4, and *z*IL-13 were significantly up-regulated in zebrafish injected with PC and MM-MSC compared to control (Fig. 4c), revealing a Th2 response.

### Effect of TLR4 inhibition on pro-tumor behavior of MSC

**Fig. 5:**
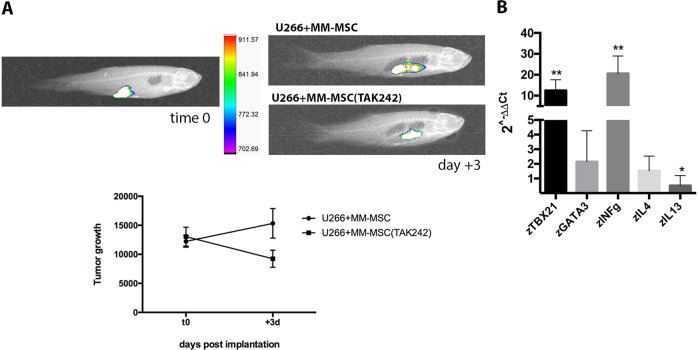
Inhibition of TLR4 signaling in MM-MSC decreased PC engraftment in vivo. MM-MSC (from five patients) were pre-treated with TAK-242 before co-injection with PC. Three animals were engrafted for every group. **a** Evaluation of tumor xenograft was performed by tomography after 3 days. Tumor growth within 3 days of engraftment in animals injected with PC + MM-MSC: *p* < 0.05; tumor reduction within 3 days in animals injected with PC + MM-MSC pretreated with TAK-242 5 μM: *p* < 0.01. Difference between the two groups: *p* < 0.001. **b** Analysis of changes in the Th1/Th2 balance in zebrafish. *p < 0.05; **p < 0.01

**Fig. 6:**
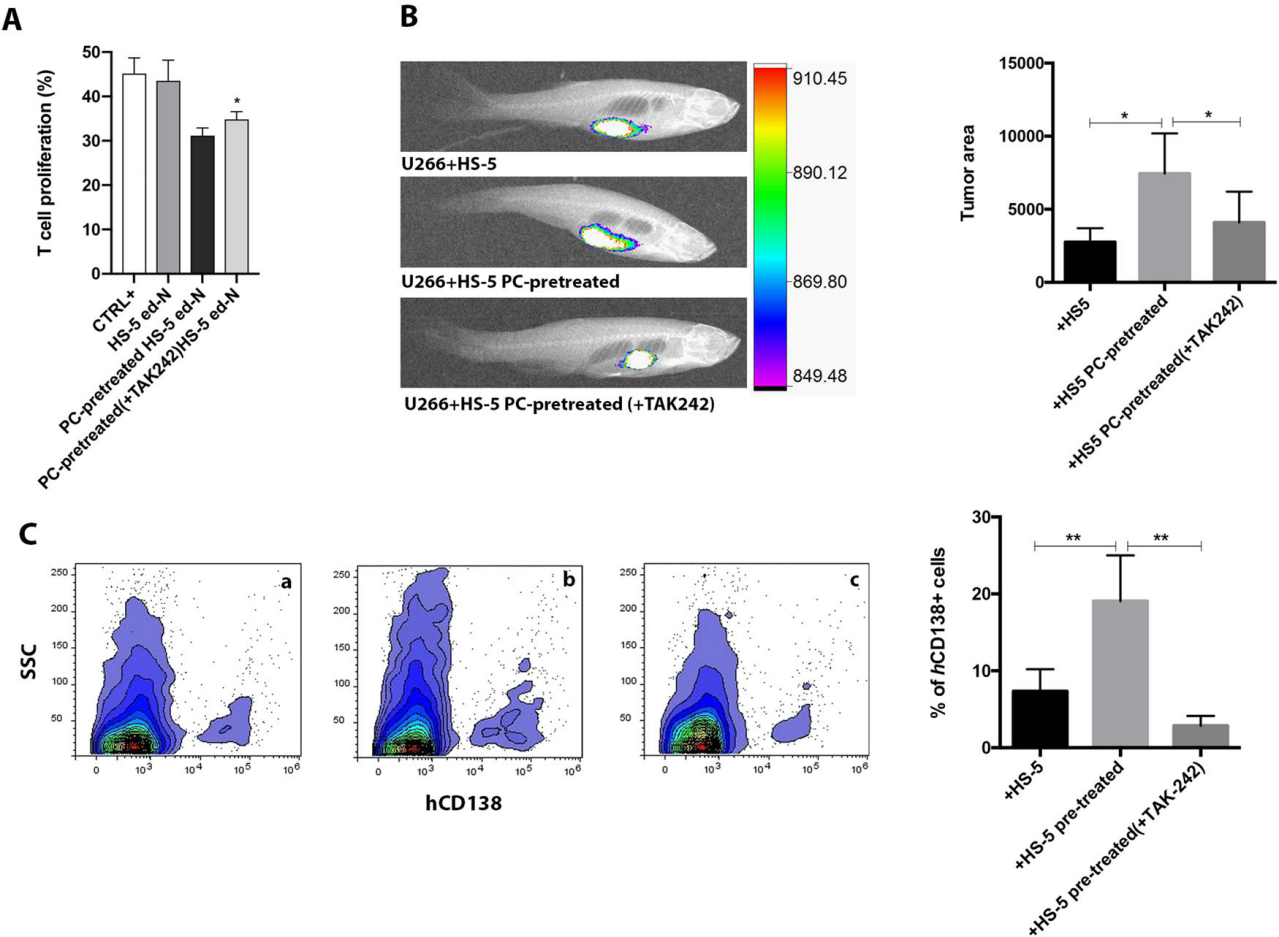
Effects of the inhibition of TLR4 during co-culture in vitro of HS-5 with MM cell lines (U266, OPM2, H929) before injection in zebrafish. **a** Evaluation of inhibitory capacity of ed-N obtained from coculture of HS-5 pre-treated with MM cells in presence or not of TAK-242. **b** Evaluation of tumor xenograft was performed by tomography after 3 days. Eight animals were engrafted for every group. (**c**) *h*CD138 infiltrate was detected by using flow cytometry. **a** U266 + HS-5; **b** U266 + HS-5 pre-treated; **c** U266 + HS-5 pre-treated (+TAK-242). *p < 0.05; **p < 0.01

To determine the role of TLR4 in MM-MSC tumor-promoting phenotype, we blocked its signaling by addition of TAK-242. MM-MSC were pre-treated for 2 h with TAK-242 before co-injection with MM cells in zebrafish. Animals injected with PC and MM-MSC pre-treated with TAK-242 showed 41% less tumor engraftment (*p* < 0.001) (Fig. 5a). We next evaluated in vivo the effects of TAK-242 pre-treatment on Th1/Th2 balance. Our results showed that zebrafish co-injected with PC and MM-MSC pre-treated with TAK-242 significantly up-regulated *z*TBX21 and *z*INFγ and down-regulated *z*IL-13, revealing a Th1 response (Fig. 5b). TAK-242 was also used to demonstrate that PC activate healthy MSC in pro-tumor stromal cells through a pro-inflammatory phenotype mediated by TLR4 signaling. Therefore, HS-5 cells were incubated with MM cell lines for 24 h in presence or not of TAK-242. After washing, these PC-educated MSC were co-cultured in vitro with PBMC or were co-injected with U266 cells in vivo. Inhibition of TLR4 pathway during co-culture of HS-5 with MM cell lines partially inhibited the ability of these PC-educated MSC to activate in vitro immature neutrophils in immunosuppressive cells (*p* < 0.05) (Fig. 6a). In vivo, animals injected with U266 and PC pre-treated HS-5 showed more myeloma tumor engraftment (*p* < 0.05) (Fig. 6b) and 17.9 ± 7% more *h*CD138 (*p* < 0.01) compared to zebrafish injected with U266 cells and HS-5 (control group) (Fig. 6b). The addition of TAK-242 during co-culture in vitro prevented the acquisition of pro-tumor ability in HS-5 after PC exposure. Indeed, by injecting U266 cells with HS-5 educated by PC in presence of TAK-242, we observed a reduction of tumor growth (*p* < 0.05; Fig. 6c) and *h*CD138 infiltrate (from 17.9 ± 7% to 2.8 ± 1.2%; *p* < 0.01; Fig. 6b).

## Discussion

Our experiments confirmed the importance of MSC in the pathogenesis of MM, providing evidence of a clear functional difference between MSC from HC or MGUS patients on one side and MSC from SMM or MM patients on the other. Indeed, similarly to MM-MSC^18^, SMM-MSC were able to modulate neutrophils in their differentiation process creating a functional polarization state that promotes immune escape and tumor growth. SMM is the intermediate asymptomatic stage distinguished from MGUS by a much higher risk of progression to MM. Since the current study clearly shows that the immunological dysfunction in MSC compartment appears already in SMM patients, the activation of an immunosuppressive microenvironment directed by MSC may contribute to the transition from MGUS to MM supporting the idea that MSC “transformation” might represent an evolutionary advantage acquired during the multistep development of cancer. In addition to MSC, also neutrophils constitute an important portion of the infiltrating immune cells in the tumor microenvironment. Neutrophils accompanying tumors are often referred to as tumor-associated neutrophils (TAN). They can exhibit an anti-tumor (N1) or a pro-tumor (N2) phenotype. The majority of TAN appear to have a N2 phenotype recruiting immunosuppressive Treg and highly expressing arginase1 responsible of T lymphocytes inactivation and releasing high levels of ROS inducing T cells apoptosis^34^. N2-polarized neutrophils also stimulate angiogenesis by releasing a number of molecules activating endothelial cells^35,36^. Immunosuppressive and pro-angiogenic neutrophils have also been found in myeloma patients displaying an immature or activated phenotype and are generally defined as granulocytic myeloid-derived suppressor cells (G-MDSC)^18,37,38^. The immunosuppressive function exerted by G-MDSC is mainly defined according to their ability to suppress T-cell activation/proliferation primarily mediated by overproduction of arginase 1^39,40^. In myeloma, as well as in chronic myeloid leukemia^41^, mature neutrophils also display an immunosuppressive ability accompanied with a change in nuclei morphology of the and longer survival in vitro^42^. The current study demonstrates that immature neutrophils cultured with SMM- or MM-MSC acquired a N2 phenotype and were able to inhibit T cells proliferation and induce angiogenesis in vitro. Moreover, only SMM- and MM-MSCed-N significantly upregulated arginase1 and NOS2, whose co-expression provides a molecular marker of immunosuppressive neutrophils^43^ and TNFα, that arrests differentiation of immature myeloid cells increasing their suppressive activity^44^. Drugs commonly used in the treatment of MM are able to target not only tumor cells but also the BM microenvironment and therefore, we also evaluated whether bortezomib, lenalidomide and pomalidomide modulate MM-MSC-mediated neutrophils activation. Interestingly, we showed that pharmacological treatments in MM-MSC and PBMC co-culture led neutrophils to lose their pro-angiogenic capacity but not their immunosuppressive ability.

We further demonstrated that myeloma PC drive MSC commitment to support tumor progression. Indeed, after pretreatment with MM cell lines, HC-MSC showed the same functional activity observed for SMM- and MM-MSC by switching healthy neutrophils in N2-polarized cells. Previous studies have suggested that MSC may acquire proinflammatory or anti-inflammatory activities depending on the stimuli received from the microenvironment. Such MSC duality of function has been compared to the polarization of macrophages with a “licensing” process depending on the TLR ligand used for activation. In particular, stimulation of TLR4 or TLR3 on BM-MSC generates pro-inflammatory MSC1 or immunosuppressive MSC2^45^. As far as concern the downstream signaling of TLR4 and TLR3 in HC-MSC after PC exposure, we observed both NF-κB and IRF3 nuclear translocation. In particular, TLR4 and TLR3 pathways share the same downstream pathway^46^. TLR4 signaling involves both MyD88-dependent and MyD88-independent pathways, activating respectively NF-κB pathway and induction of pro-inflammatory cytokines, and IRF3 pathway responsible for type I IFN production^47^. TLR3-mediated signaling is MyD88-independent and it requires TIR domain-containing adapter inducing IFNβ to activate IRF3. However, stimulation of TLR3 for 24 h also leads to the activation of NF-κB^48,49^. Therefore, to furthter define which TLR signaling is involved in immunological altered behavior of MM-MSC, we pre-treated HC-MSC with LPS or poly(I:C) as agonist of TLR4 and TLR3 respectively. Only LPS exposure was able to switch HC-MSC in “activated” stromal cells, suggesting that change in immunological phenotype of MM-MSC may be linked to a pro-inflammatory phenotype mediated by TLR4 signaling. Consistently with our results, Lu et al. demonstrated that TLR4 + MSC from acute myeloid leukemia and lung cancer patients suppressed NK cell proliferation and this inhibitory effect was enhanced by the activation of the cells following LPS treatment^17^.

We also investigated TLR4 expression in MSC from MM patients observing no significant differences compared to HC-MSC. However, MM-MSC over-expressed MyD88, suggesting the activation of TLR4 signaling. To better investigate the role of TLR4 in pro-tumor activity of MM-MSC, we used the adult zebrafish as in vivo model of MM engraftment. This model has some advantages over the classic mouse model. The limitations of xenograft models of human myeloma in mice includes the need for human fetal bone fragments to support the tumor growth of PC obtained from patients, the low efficiency resulting in the generation of a limited number of tumor-bearing mice and two months for tumor growth^29^. Their small body size, high fecundity, swift extracorporeal embryogenesis, and transparency of their bodies during development have facilitated the use of zebrafish in genetic and live-imaging studies^50–52^. Sacco et al. developed a zebrafish embryo model for rapid screening of MM cells that can home or metastasize to the BM-like niche region, the caudal hematopoietic tissue (CHT)^53^. The authors reported that changes observed in MM cells following interaction with the CHT-niche are identical to those that are usually seen after the interaction of myeloma PC with the human BM microenvironment (enrichment for cytokine/chemokine-, angiogenesis- and adhesion–related mRNA signatures). Allowing efficient and rapid growth of MM cells, zebrafish has also been proposed as a screening platform for drug activity and for studying the biology of MM^29^. Here, we have described an adult zebrafish model of MM xenograft with the advantage of being an immunocompetent model for deciphering the mechanisms of immunity control^54^. Therefore, we concentrated our attention on pro-tumor ability of SMM- and MM-MSC in vivo co-injecting them with myeloma PC. Tomography image analysis showed a higher efficiency of tumor engraftment in animals injected with PC and SMM- or MM-MSC compared to zebrafish co-injected with PC and HC-MSC. We have also shown that it is possible to perform an accurate measurement of human MM cell percentage in zebrafish by using flow cytometry. The obtained results confirmed tomography data revealing more *h*CD138+ in zebrafish co-injected with PC cells and SMM- or MM-MSC. The immune system of zebrafish resembles in several aspects the mammalian immune system^55,56^. Therefore, since Th1 (tbx21 and INFγ1-2) and Th2 signature genes (gata3 and IL4) are evolutionarily conserved in zebrafish, we used this model to understand in vivo if co-injection of PC with MM-MSC favors immune escape mechanisms. The increased expression of the Th2-type cytokines (*z*IL-4 and *z*IL-13) in the animals injected with PC and MM-MSC was consistent with the up-regulation of *z*GATA3, the master regulatory transcription factor for Th2 lineage development^56^, suggesting a Th2 response. Considering the linking role between TLR4 signaling and “activated” status of HC-MSC resembling MM-MSC behavior, we investigated whether TAK-242 affects pro-tumor capacity of MM-MSC in vivo. This molecule binds selectively to TLR4 and subsequently disrupts the interaction of TLR4 with adaptor molecules, thereby inhibiting the signal transduction and its downstream signaling events^57^. By using TAK-242 before co-injection with PC, tumor engraftment significantly decreased with an up-regulation of TBX21 and INFγ and a consequent Th1 response. Additionally, we used TAK-242 also during phenotype activation of HS-5 by PC in order to demonstrate that MM cells activate in healthy MSC a pro-inflammatory status to better serve cancer. In vitro experiments demonstrated that inhibition of TLR4 signaling during co-culture of HS-5 with MM cell lines partially decreased the ability of PC-pretreated HS-5 to educate immature neutrophils in immunosuppressive cells. Next, animals were injected with PC and HS-5 cells pretreated with MM cell lines in presence or not of TAK-242. Zebrafish injected with U266 cells and PC-pretreated HS-5 showed more tumor engraftment and more *h*CD138 compared to animals injected with U266 cells and HS-5 (control group). On the contrary, injection of U266 cells with HS-5 pre-treated with PC in presence of TAK-242 led to a lower tumor growth and *h*CD138 infiltrate.

In conclusion, our work identifies TLR4 as an important pro-tumor activator of MSC phenotype suggesting that upon activation, the stroma is progressively inflammatory-imprinted by PC to an “autonomous” state where it becomes independent from cancer cells because MM-MSC maintain this immune alteration after in vitro expansion. Therefore, during the advanced stage of the disease, this inflammatory vicious circle may become difficult to break without combined stroma-targeting therapies. Unravelling the mechanisms by which MSC contributes and responds to the inflammatory process would therefore help in the development of new immune-based therapies directed to tumor cells eradication.

## Supplementary information

supplementary material

## References

[1] Palumbo A, Anderson K (2011). Multiple myeloma. N. Engl. J. Med..

[2] Kristinsson SY, Landgren O, Dickman PW, Derolf AR, Bjorkholm M (2007). Patterns of survival in multiple myeloma: a population-based study of patients diagnosed in Sweden from 1973 to 2003. J. Clin. Oncol..

[3] Kawano Y (2015). Targeting the bone marrow microenvironment in multiple myeloma. Immunol. Rev..

[4] Desterke C, Martinaud C, Ruzehaji N, Le Bousse-Kerdiles MC (2015). Inflammation as a keystone of bone marrow stroma alterations in primary myelofibrosis. Mediators Inflamm..

[5] Graham Nicola, Qian Bin-Zhi (2018). Mesenchymal Stromal Cells: Emerging Roles in Bone Metastasis. International Journal of Molecular Sciences.

[6] Zambetti NA (2016). Mesenchymal inflammation drives genotoxic stress in hematopoietic stem cells and predicts disease evolution in human pre-leukemia. Cell Stem Cell.

[7] Mathew E (2016). Mesenchymal stem cells promote pancreatic tumor growth by inducing alternative polarization of macrophages. Neoplasia.

[8] Karnoub AE (2007). Mesenchymal stem cells within tumour stroma promote breast cancer metastasis. Nature.

[9] Xu WT, Bian ZY, Fan QM, Li G, Tang TT (2009). Human mesenchymal stem cells (hMSCs) target osteosarcoma and promote its growth and pulmonary metastasis. Cancer Lett..

[10] Torsvik A, Bjerkvig R (2013). Mesenchymal stem cell signaling in cancer progression. Cancer Treat. Rev..

[11] Patel SA (2010). Mesenchymal stem cells protect breast cancer cells through regulatory T cells: role of mesenchymal stem cell-derived TGF-beta. J. Immunol..

[12] Nakamura K (2018). Dysregulated IL-18 is a key driver of immunosuppression and a possible therapeutic target in the multiple myeloma microenvironment. Cancer Cell.

[13] Nakamura K, Smyth MJ (2017). Targeting cancer-related inflammation in the era of immunotherapy. Immunol. Cell Biol..

[14] Akira S (2003). Toll-like receptor signaling. J. Biol. Chem..

[15] Tomchuck SL (2008). Toll-like receptors on human mesenchymal stem cells drive their migration and immunomodulating responses. Stem cells.

[16] Waterman RS, Henkle SL, Betancourt AM (2012). Mesenchymal stem cell 1 (MSC1)-based therapy attenuates tumor growth whereas MSC2-treatment promotes tumor growth and metastasis. PLoS ONE.

[17] Lu Y (2015). TLR4 plays a crucial role in MSC-induced inhibition of NK cell function. Biochem Biophys. Res Commun..

[18] Giallongo C (2016). Granulocyte-like myeloid derived suppressor cells (G-MDSC) are increased in multiple myeloma and are driven by dysfunctional mesenchymal stem cells (MSC). Oncotarget.

[19] Giallongo C (2015). Myeloid derived suppressor cells in chronic myeloid leukemia. Front Oncol..

[20] Giallongo C (2016). Mesenchymal stem cells (MSC) regulate activation of granulocyte-like myeloid derived suppressor cells (G-MDSC) in chronic myeloid leukemia patients. PLoS ONE.

[21] Lechner MG (2011). Functional characterization of human Cd33+ and Cd11b+ myeloid-derived suppressor cell subsets induced from peripheral blood mononuclear cells co-cultured with a diverse set of human tumor cell lines. J. Transl. Med.

[22] Murray MY (2015). Ibrutinib inhibits BTK-driven NF-kappaB p65 activity to overcome bortezomib-resistance in multiple myeloma. Cell Cycle.

[23] Munemasa S (2008). Osteoprogenitor differentiation is not affected by immunomodulatory thalidomide analogs but is promoted by low bortezomib concentration, while both agents suppress osteoclast differentiation. Int J. Oncol..

[24] Papandreou CN (2004). Phase I trial of the proteasome inhibitor bortezomib in patients with advanced solid tumors with observations in androgen-independent prostate cancer. J. Clin. Oncol..

[25] Lupo G (2014). An in vitro retinoblastoma human triple culture model of angiogenesis: a modulatory effect of TGF-beta. Cancer Lett..

[26] Giallongo C (2011). BRIT1/MCPH1 expression in chronic myeloid leukemia and its regulation of the G2/M checkpoint. Acta Haematol..

[27] Vittori M (2016). Imaging of human glioblastoma cells and their interactions with mesenchymal stem cells in the zebrafish (Danio rerio) embryonic brain. Radio. Oncol..

[28] Frassanito MA (2014). Bone marrow fibroblasts parallel multiple myeloma progression in patients and mice: in vitro and in vivo studies. Leukemia.

[29] Lin J (2016). A clinically relevant in vivo zebrafish model of human multiple myeloma to study preclinical therapeutic efficacy. Blood.

[30] Gupta, T. & Mullins, M. C. Dissection of organs from the adult zebrafish. *J. Vis. Exp*., 10.3791/1717 (2010). 10.3791/1717 PMC3144575 20203557

[31] Iwanami N (2014). Zebrafish as a model for understanding the evolution of the vertebrate immune system and human primary immunodeficiency. Exp. Hematol..

[32] Zhu LY, Pan PP, Fang W, Shao JZ, Xiang LX (2012). Essential role of IL-4 and IL-4Ralpha interaction in adaptive immunity of zebrafish: insight into the origin of Th2-like regulatory mechanism in ancient vertebrates. J. Immunol..

[33] Hammaren MM (2014). Adequate Th2-type response associates with restricted bacterial growth in latent mycobacterial infection of zebrafish. PLoS Pathog..

[34] Lu T, Gabrilovich DI (2012). Molecular pathways: tumor-infiltrating myeloid cells and reactive oxygen species in regulation of tumor microenvironment. Clin. Cancer Res.

[35] Nozawa H, Chiu C, Hanahan D (2006). Infiltrating neutrophils mediate the initial angiogenic switch in a mouse model of multistage carcinogenesis. Proc. Natl Acad. Sci. USA.

[36] Zhang X (2016). Neutrophils in cancer development and progression: roles, mechanisms, and implications (Review). Int J. Oncol..

[37] Gorgun GT (2013). Tumor-promoting immune-suppressive myeloid-derived suppressor cells in the multiple myeloma microenvironment in humans. Blood.

[38] Binsfeld M (2016). Granulocytic myeloid-derived suppressor cells promote angiogenesis in the context of multiple myeloma. Oncotarget.

[39] Romano A (2014). Immunological dysregulation in multiple myeloma microenvironment. Biomed. Res. Int..

[40] Romano A (2018). PMN-MDSC and arginase are increased in myeloma and may contribute to resistance to therapy. Expert Rev. Mol. Diagn..

[41] Giallongo C (2014). Myeloid derived suppressor cells (MDSCs) are increased and exert immunosuppressive activity together with polymorphonuclear leukocytes (PMNs) in chronic myeloid leukemia patients. PLoS ONE.

[42] Puglisi F (2019). Plasticity of high-density neutrophils in multiple myeloma is associated with increased autophagy via STAT3. Int. J. Mol. Sci..

[43] Talmadge JE (2007). Pathways mediating the expansion and immunosuppressive activity of myeloid-derived suppressor cells and their relevance to cancer therapy. Clin. Cancer Res.

[44] Sade-Feldman M (2013). Tumor necrosis factor-alpha blocks differentiation and enhances suppressive activity of immature myeloid cells during chronic inflammation. Immunity.

[45] Waterman RS, Tomchuck SL, Henkle SL, Betancourt AM (2010). A new mesenchymal stem cell (MSC) paradigm: polarization into a pro-inflammatory MSC1 or an Immunosuppressive MSC2 phenotype. PLoS ONE.

[46] Delarosa O, Dalemans W, Lombardo E (2012). Toll-like receptors as modulators of mesenchymal stem cells. Front. Immunol..

[47] Lu YC, Yeh WC, Ohashi PS (2008). LPS/TLR4 signal transduction pathway. Cytokine.

[48] Jiang Z, Mak TW, Sen G, Li X (2004). Toll-like receptor 3-mediated activation of NF-kappaB and IRF3 diverges at Toll-IL-1 receptor domain-containing adapter inducing IFN-beta. Proc. Natl Acad. Sci. USA.

[49] Dumitru CA, Hemeda H, Jakob M, Lang S, Brandau S (2014). Stimulation of mesenchymal stromal cells (MSCs) via TLR3 reveals a novel mechanism of autocrine priming. FASEB J..

[50] Lieschke GJ, Currie PD (2007). Animal models of human disease: zebrafish swim into view. Nat. Rev. Genet.

[51] Stainier DY (2001). Zebrafish genetics and vertebrate heart formation. Nat. Rev. Genet.

[52] Howe K (2013). The zebrafish reference genome sequence and its relationship to the human genome. Nature.

[53] Sacco A (2016). Cancer cell dissemination and homing to the bone marrow in a zebrafish model. Cancer Res.

[54] Luukinen Hanna, Hammarén Milka Marjut, Vanha-aho Leena-Maija, Svorjova Aleksandra, Kantanen Laura, Järvinen Sampsa, Luukinen Bruno Vincent, Dufour Eric, Rämet Mika, Hytönen Vesa Pekka, Parikka Mataleena (2017). Priming of innate antimycobacterial immunity by heat-killed Listeria monocytogenes induces sterilizing response in the adult zebrafish tuberculosis model. Disease Models & Mechanisms.

[55] Quintana FJ (2010). Adaptive autoimmunity and Foxp3-based immunoregulation in zebrafish. PLoS ONE.

[56] Sugimoto K, Hui SP, Sheng DZ, Nakayama M, Kikuchi K (2017). Zebrafish FOXP3 is required for the maintenance of immune tolerance. Dev. Comp. Immunol..

[57] Matsunaga N, Tsuchimori N, Matsumoto T, Ii M (2011). TAK-242 (resatorvid), a small-molecule inhibitor of Toll-like receptor (TLR) 4 signaling, binds selectively to TLR4 and interferes with interactions between TLR4 and its adaptor molecules. Mol. Pharm..

