# Correction: TLR4 signaling drives mesenchymal stromal cells commitment to promote tumor microenvironment transformation in multiple myeloma

**DOI:** 10.1038/s41419-019-2059-2

**Published:** 2019-10-28

**Authors:** Cesarina Giallongo, Daniele Tibullo, Giuseppina Camiolo, Nunziatina L. Parrinello, Alessandra Romano, Fabrizio Puglisi, Alessandro Barbato, Concetta Conticello, Gabriella Lupo, Carmelina Daniela Anfuso, Giacomo Lazzarino, Giovanni Li Volti, Giuseppe Alberto Palumbo, Francesco Di Raimono

**Affiliations:** 1 Section of Haematology, Department of General Surgery and Medical-Surgical Specialties, University of Catania, Catania, Italy; 2 Department of Biomedical and Biotechnological Sciences, University of Catania, Catania, Italy; 3 EuroMediterranean Institute of Science and Technology, Palermo, Italy; 4 Institute of Biochemistry and Clinical Biochemistry, Catholic University of Rome, Largo F. Vito 1, 00168 Rome, Italy; 5 Department of Medical, Surgical Sciences and Advanced Technologies “G. F. Ingrassia”, University of Catania, Catania, Italy

**Keywords:** Myeloma, Translational research

**Correction to: Cell Death and Disease**

10.1038/s41419-019-1959-5; published online 20 September 2019

Following publication of this article, it was noted that Francesco Di Raimondo’s name had been listed incorrectly as “Francesco Di Raimono”. This has been corrected in the both the PDF and HTML versions of the Article.

