# Supplementary material for: TLR4 signaling drives mesenchymal stromal cells commitment to promote tumor microenvironment transformation in multiple myeloma
Source: Cell Death Dis. 2019 Sep 20;10(10):704. doi: 10.1038/s41419-019-1959-5 (PMC6754430; doi:10.1038/s41419-019-1959-5)
Supplement: Supplementary file 1 — supplementary material [file 41419_2019_1959_MOESM1_ESM.doc]

**Supplementary methods**

**Materials**

**Flow cytometry**

After separation magnetic cell separation, educated neutrophils were incubated with fluorescently labeled anti-CD16 PC5 (clone 3G8) and anti-CD66b FITC (clone 80H3) antibodies (Beckman coulter, Brea, California, United States) to evaluate their purity by using flow cytometry. The appropriate isotopic control was also included. Labeled samples were acquired using a Beckman Coulter FC-500 flow cytometer (Beckman Coulter FC-500 flow cytometer).

**XTT assay**

MM-MSC and HS5 cells (1x10^4^) were seeded in a 96-well plate and incubated with different concentration of TAK-242. After 24h the XTT cell viability kit (Cell signaling tech. 9095) was added to the plate and cells were incubated for 5h. The absorbance at 450 nm was measured.

**Supplementary figures**


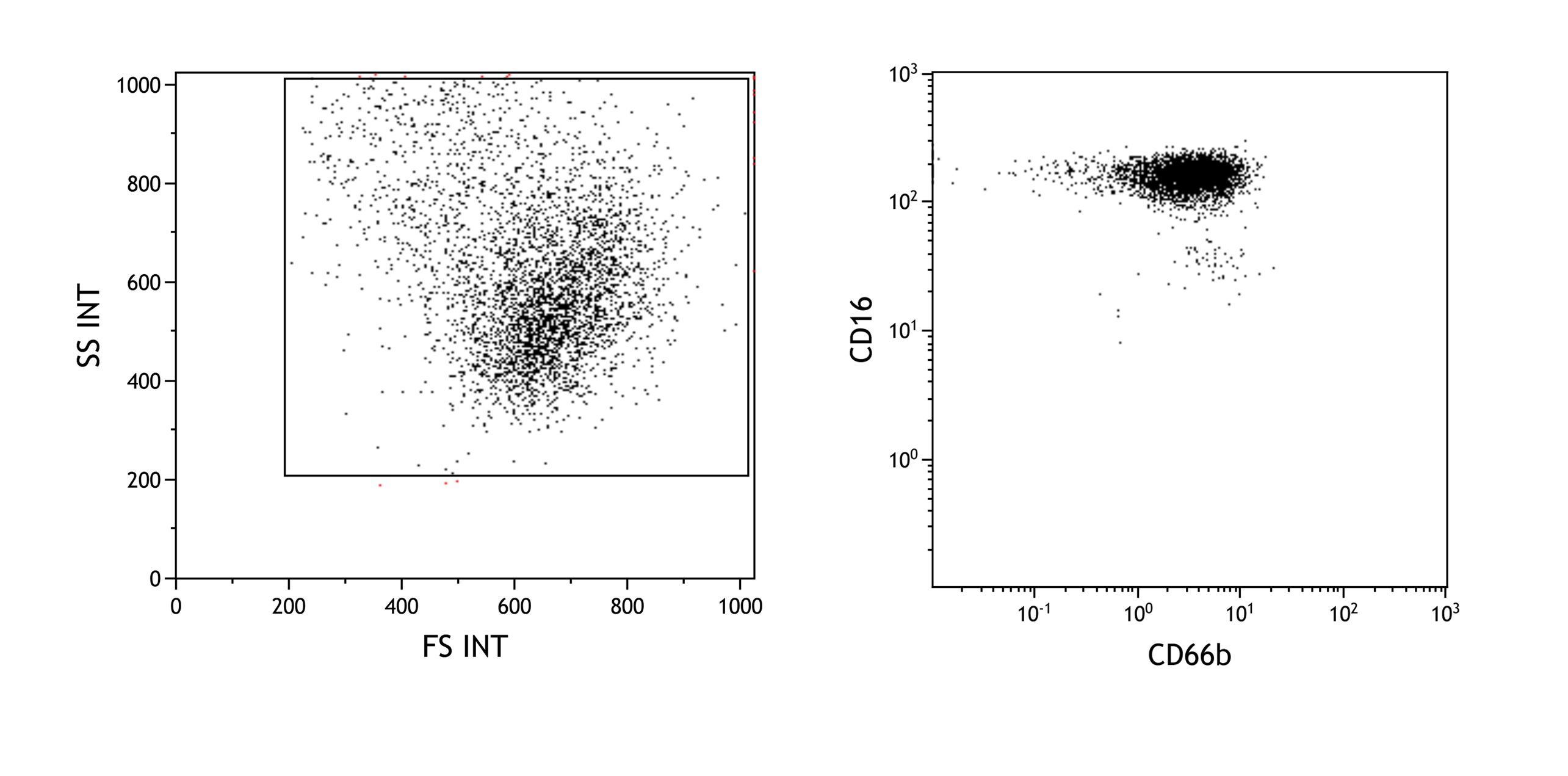


**Supplementary Figure 1.** Purity of educated Neutrophils after magnetic cell separation. The figure reports the representative flow cytometry dot plots showing the purity of educated Neutrophils.


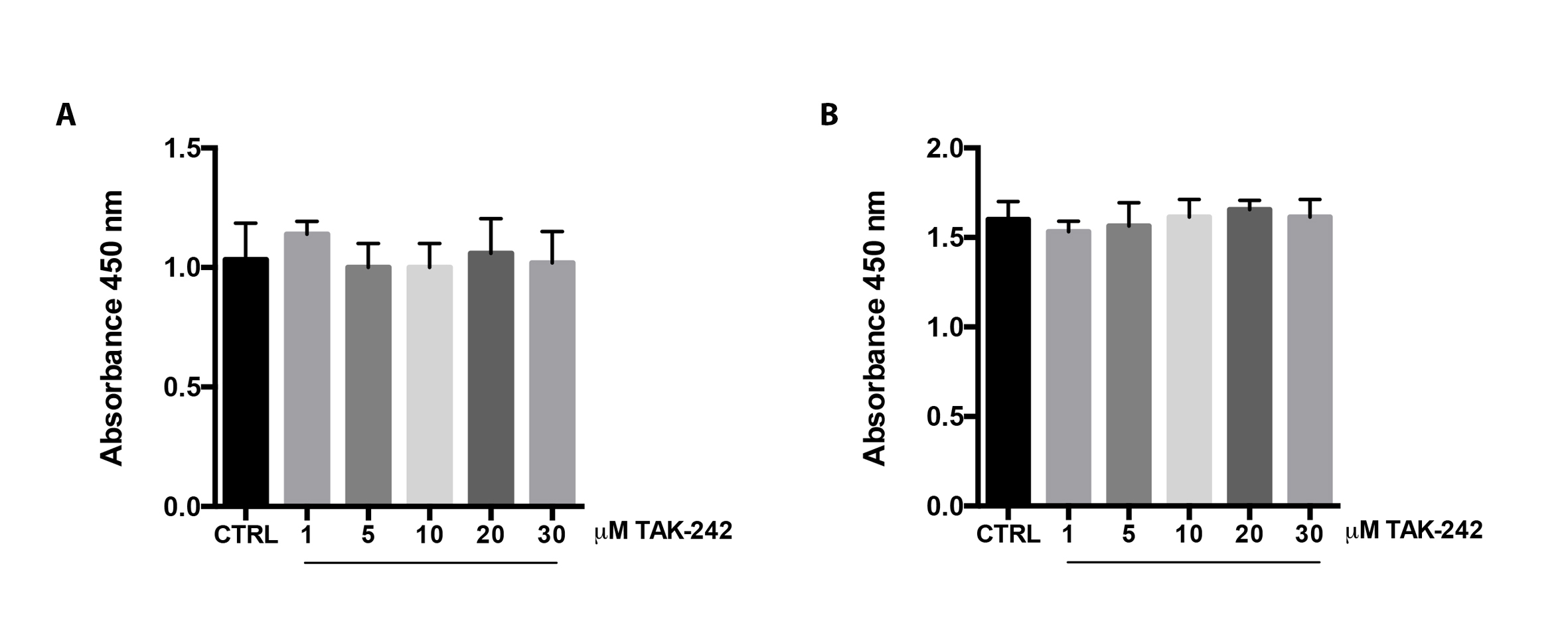


**Supplementary Figure 2.** TAK-242 does not affect primary MSC or HS-5 cells viability in vitro. MSC from 3 MM patients (A) and HS-5 cell lines were cultured with different doses of TAK-242. After 24h, XTT assay was used to determine cell viability.

**
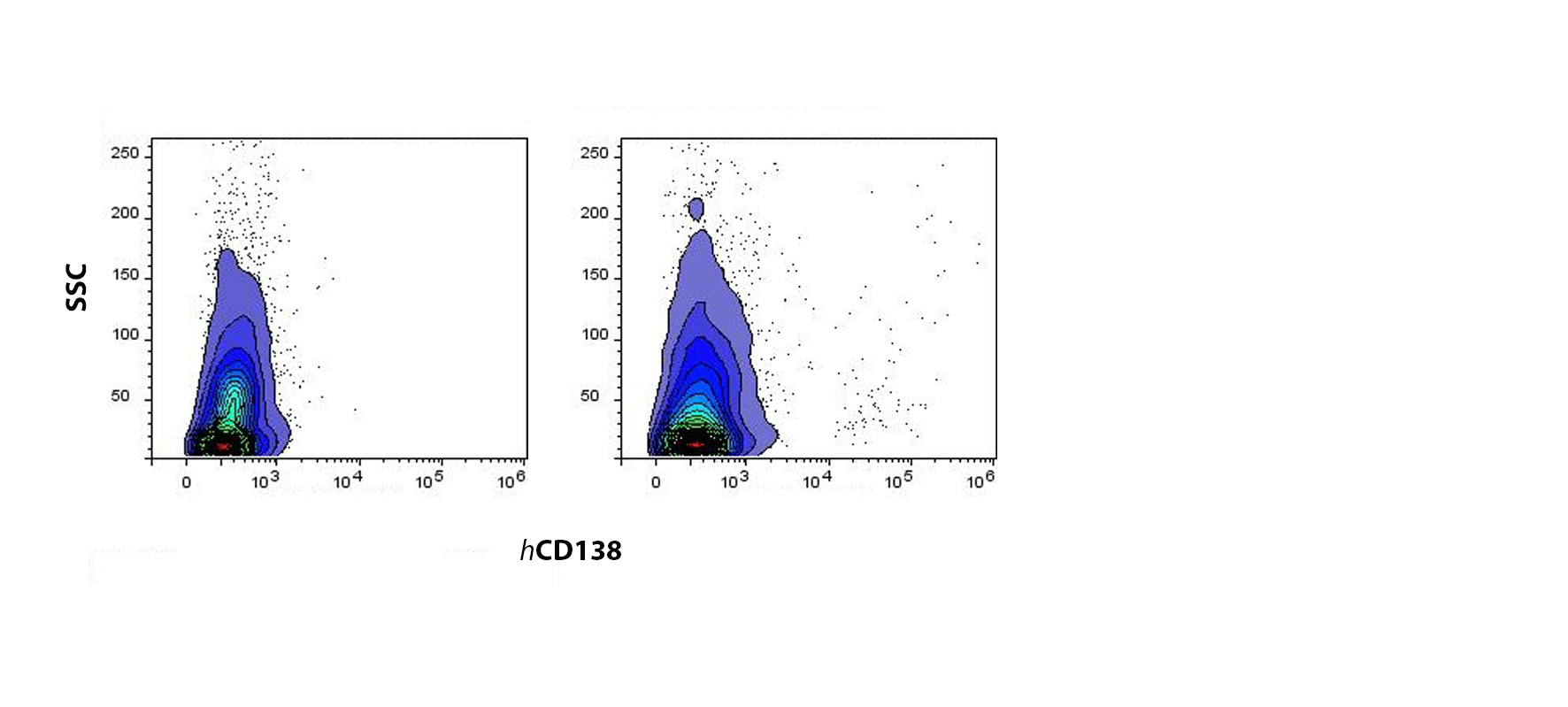
**

**Supplementary figure 3.** Side scatter vs *h*CD138 plots showed no differences in *h*CD138 binding between zebrafish sample labeled with MoAb *h*CD138 (right panel) and unstained control (left panel), confirming that anti human monoclonal CD138 showed no crossreaction with zebrafish cells.

**Supplementary figure 4.** Primers and accession numbers of target genes.

**Zebrafish (*Danio* rerio)**

*Tbx21* ZDB-GENE-080104-3 F: GGCCTACCAGAATGCAGACA, R: GGTGCGTACAGCGTGTCATA

*Gata3* ZDB-GENE-990415-82 F: GGATGGCACCGGTCACTATT, R: CAGCAGACAGCCTCCGTTT

*IL4* ZDB-GENE-100204-1 F: GCAGGAATGGCTTTGAAGGG R: GCAGTTTCCAGTCCCGGTAT

*IL13* ZDB-GENE-100727-2 F: GGAAGCTGTGTTAGTCAATCC R: GCCTGACAGAAATAATCATGC

*IFNγ1-2* ZDB-GENE-040629-1 F: GGGCGATCAAGGAAAACGACCC, R: TAGCCTGCCGTCTCTTGCGT

*GAPDH* ZDB-GENE-030115-1 F: AGTGTCAGGACGAACAGAGGCT,R:GCCAATGCGACCGAATCCGTTA
